# Patient-centred access to health care: a framework analysis of the care interface for frail older adults

**DOI:** 10.1186/s12877-018-0960-7

**Published:** 2018-11-12

**Authors:** Donata Kurpas, Holly Gwyther, Katarzyna Szwamel, Rachel L. Shaw, Barbara D’Avanzo, Carol A. Holland, Maria Magdalena Bujnowska-Fedak

**Affiliations:** 1 Department of Family Medicine, Wrocław Medical University, ul. Syrokomli 1, 51-141 Wrocław, Poland; 2 Opole Medical School, ul. Katowicka 68, 45-060 Opole, Poland; 3 Centre For Ageing Research, Lancaster University, Lancaster, UK; 4 Psychology, School of Life & Health Sciences, Aston University, Birmingham, UK; 5 Istituto di Ricerche Farmacologiche Mario Negri IRCCS, Milan, Italy

**Keywords:** Frailty, Delivery of health care, Health resources, Patient acceptance of health care, Patient preference, Patient satisfaction

## Abstract

**Background:**

The objective of this study was to explore the issues surrounding access to health and social care services for frail older adults with Polish stakeholders, including healthy and frail/pre-frail older adults, health care providers, social care providers, and caregivers, in order to determine their views and perspectives on the current system and to present suggestions for the future development of a more accessible and person-centred health and social care system.

**Methods:**

Focus groups were used to gather qualitative data from stakeholders. Data were analysed using framework analysis according to five dimensions of accessibility to care: approachability, acceptability, availability and accommodation, affordability and appropriateness.

**Results:**

Generally services were approachable and acceptable, but unavailable. Poor availability related to high staff turnover, staff shortages and a lack of trained personnel. There were problems of long waiting times for specialist care and rehabilitation services, and geographically remote clinics. Critically, there were shortages of long-term inpatient care places, social care workers and caregivers. The cost of treatments created barriers to care and inequities in the system. Participants described a lack of integration between health and social care systems with differing priorities and disconnected budgets. They described an acute medical system that was inappropriate for patients with complex needs, alongside a low functioning social care system, where bureaucratisation caused delays in providing services to the vulnerable. An integrated system with a care coordinator to improve connections between services and patients was suggested.

**Conclusions:**

There is an immediate need to improve access to health and social care systems for pre-frail and frail patients, as well as their caregivers. Health and social care services need to be integrated to reduce bureaucracy and increase the timeliness of treatment and care.

## Background

Equity in health and social care is critical in realising the full health potential of a population. Evidence shows that there are disparities in the quality of health and health care within and between European Union (EU) member states [1]. These disparities can be conceptualised as population-specific differences in the presence of disease, health outcomes, and access to health care, but can also be seen in terms of life expectancy and healthy life years [2]; that is, the number of years lived in good health as an indicator of quality of life rather than longevity.

Health and social care disparities are caused by a range of social determinants including socio-economic policy, environmental characteristics, poverty, unemployment levels, and the organisation and functioning of the health and welfare systems; individual factors such as lifestyle choices, age, and health behaviours are also critical [3] and may also vary between cultures. It is generally accepted that health care inequalities are “unfair and unjust” (p29. [4]) and thus, reducing them is an ethical imperative. In 2005, all EU member states committed to reducing inequalities for access to health care.

Population ageing has many socio-economic and health consequences, but one challenge is the need to build effective and accessible health and social care systems for older adults, as well as appropriate support networks for their families, whilst balancing budgetary constraints [5, 6]. Facilitating access to health and social care is an important step in enabling people to preserve or improve their health but using such services requires effort, and vulnerable groups of people, for example, those who are older or socially deprived, may not have the appropriate range of knowledge-based, social, language or practical skills to mobilise that effort [7].

Within the older adult population, frail people may be particularly vulnerable. Most frail individuals suffer from chronic diseases, with a statistically significant increase in frailty in people with a greater number of co-morbidities [8, 9]. Indeed, the number of chronic diseases is an important constituent part of the calculation of a frailty score, using an accumulation of deficits model of frailty [10]. Such multi-morbidity relates to elevated rates of primary care and multiple specialist visits. Communication between the array of health care providers can be poorly coordinated or lacking, which subsequently affects health care costs, patient outcomes, and experiences of care [11]. In order to formulate effective and enduring care plans for these vulnerable patients, a range of factors must be addressed, including appropriate access to health care, care coordination and improved communication between stakeholders in older adult care, including caregivers [12, 13].

Many caregivers take on all-consuming roles as intermediaries between the health and social care services and their care recipient. In essence, they take on the ‘effort’ of using services and assume the role of the active participant on behalf of their older relative [14]. However, evidence suggests that these advocates often have poor experiences of the health care system, finding it fragmented, rigid, and difficult to access [13, 15]. In this era of changing health care priorities, an ageing population, and the growing burden of chronic diseases, understanding the challenges faced by caregivers is crucial when designing suitable support services for them within both health care and community settings [16].

It should be emphasised here that treating frailty in older adults is a realistic therapeutic goal [17] and previous interventions have been shown to be effective [18]. One definition suggests that frailty is a dynamic process characterised by frequent transitional stages, which can be modified [19, 20]. This conceptualisation of frailty as a treatable entity may provide new opportunities for prevention, management, and improved care at both the population and clinical level [9] opportunities which are not currently being exploited by all health and social care organisations.

Certainly in Poland, there is a view that the organisation of the health and social care systems are ill-prepared and under-equipped to meet the needs of the growing population of frail older adults. Since 1991, there has been a progressive disintegration of the health and social care systems including division into two separately administered and managed ministeries. This division of care has resulted in difficulties in coordinating activities in long term care (LTC: [21]) and reduced collaboration between physicians and social care workers [22]. Further, decentralisation of the previously tax-funded national health service with a social health insurance system in 1999 resulted in a defragmented system, with sixteen regional insurance funds responsible for their own budgets and contractor provision. Although the National Health Fund was established in 2003–04 with overall responsibility for purchasing, responsibility for health is still fragmented with facility ownership, service provision and delivery, and accountability at various regional, county, municipal and national governmental levels [23].

In addition, the profile of social care has changed significantly. It is now delivered by the Ministry of Family, Labour and Social Policy whose responsibilities consist mainly of determining the rights to, and allowances for, caring benefits, rather than solving real social problems [22]. It is well established that poor social support and comorbidities are negatively associated with functional status and mortality, particularly in older patients [24]. Patients who have complex health needs require both medical and social services and support from a wide variety of providers and caregivers, and the patient-centred medical home approach offers promise as a model for providing comprehensive and coordinated care [25].

There is a view that the reintegration of health and social care services for frail older adults would enhance satisfaction, quality of life, efficiency, and health outcomes and would also decrease costs [5]. It is believed that such integrated care delivery would eliminate inefficiency and the duplication of work processes while relieving professionals of their administrative burden in favour of patient-related activities [26]. It is also perceived as the best solution to address health care-related frustrations experienced by patients with chronic conditions [27], to improve their experiences of care [28, 29] and their quality of life [30].

However, there is currently insufficient research to determine whether the current health and social care systems are effective or whether care integration would make a difference to stakeholders in older adult care, in terms of access to services or indeed, the outcomes of those services. Access to health and social care is a complex concept and is dependent on both the provision of adequate services and the absence of financial, organisational, social and cultural barriers to those services [31]. Levesque et al., [32] conceptualise access to health care across five dimensions, along with five corresponding abilities of populations: 1) approachability (ability to perceive); 2) acceptability (ability to seek); 3) availability and accommodation (ability to reach); 4) affordability (ability to pay); and 5) appropriateness (ability to engage).

Previous work with European stakeholders in three countries [12], including Polish nationals, described the need for a new kind of transparent, health and social care system which was integrated and person-centred. This paper also raised awareness of the challenges associated with access to appropriate services in the complex Polish systems and described a need for more accessible care and support for older adults as well as their caregivers.

Therefore, the aim of this study is to explore the issues surrounding access to health and social care services for frail older adults, with Polish stakeholders including frail and robust older adults, health care professionals, social care workers and family caregivers, in order to determine their views and perspectives on the current system and to present stakeholders’ suggestions for the future development of more accessible and person-centred health and social care systems.

## Method

This study forms part of a wider range of studies known collectively as FOCUS [33, 34]**.** Qualitative findings from focus groups conducted in three countries: Italy (Milan), Poland (Wroclaw) and the United Kingdom (Birmingham) have previously been reported [12]. This current paper reports a secondary analysis of data from the same study but has the specific purpose of presenting findings relating to access to, and integration of, health and social care from the Polish stakeholders only.

### Procedure

Five focus groups were facilitated by two female general practitioners (DK and MBF) with some previous experience of qualitative research. Focus groups were conducted with 44 stakeholders in the care of frail older adults including healthy older adults, frail older adults, health care professionals, social care workers and family caregivers. There were between eight and ten participants in each group. No non-participants were present. Focus groups were conducted in Polish. The sessions lasted between 60 and 90 min. Semi-structured questions, defined in advance through literature review and discussion among partners, were posed (see Table 1), including broadly, experiences and attitudes toward frailty, medical treatment, quality of life and social care. Questions were not pilot tested. The facilitators were known in a professional capacity only to the participants. No personal information was relayed about the researchers to the participants.

**Table 1:** Interview schedule

Older adults (frail)	Older adults (healthy)	Health and social care providers	Family caregivers
This project is about frailty. Can you tell me what you think of when you hear that word?			
Do you consider yourself to be frail?		Do you consider the person you are caring for to be frail?	
What does frailty mean to you?			
Taking turns, can you tell me about a typical day?		Taking turns, can you tell me about the patients you care for/work with and how you might consider them frail?	
Does anybody help you with things on a day to day basis (prompts: personal care, shopping, cleaning etc.)?			
Do you receive any formal health or social care services? If so, then what sorts of services are they?		What sorts of services do you offer patients considered to be frail?	Does the person you care for receive any formal health or social care services? If so, then what sorts of services are they?
		Do you provide any support for carers for frail older adults?	Do you receive any support as a carer for a frail older adult?
Do you think there are ways that you could have prevented yourself from becoming frail?	Do you think there’s anything you can do to prevent yourself from becoming frail?	Do you think there are ways we could prevent people from becoming frail?	Do you think there are ways that you could have prevented the person you care for becoming frail?
Have you adapted your home so you can move around it more easily?	If it became necessary do you think you would be able to adapt your home so you could move around it more easily if you became frail?		Have you adapted the living space so that the person you care for can move around more easily? Are there other things you would like to do?
Do you think more help with this should be available to you?			Do you think more help with this should be available to you?
Can you think of what led up to you becoming frail?	Do you have any chronic conditions? Do you think there is a time when you might become frail yourself? Do you have friends/relatives you would consider frail?	What do you think are the causes of frailty in the patients you work with?	Can you think of what led up to the person you care for becoming frail?
Can you identify anything you might consider a cause?	What do you think might be the possible causes of frailty?	What would you say are likely causes of frailty?	Can you identify anything you might consider a cause?
Do you need help with personal care? If so, how do you feel about this?	How would you feel if you realised you needed help with personal care?		Do you look after the personal care of the person you care for? If so, did you have experience of this before?
Do you think people providing personal care should receive any guidance or support in how to best do it?		Do you provide support for carers in the provision of personal care? Do you offer any training or guidance on how to do this?	Have you received any training or guidance on how to do it?
Do you feel that your dignity or personal safety is threatened because of your frailty/need for personal care?	Do you think your dignity or personal safety would be threatened if you received help with personal care?	Do you think the dignity or personal safety of frail older adults is threatened?	Do you feel that the dignity or personal safety of the person you care for is threatened because of their frailty?
Do you think anything else could be done to protect your dignity or personal safety?	Do you think anything could be done to protect your dignity or personal safety?	Do you think you could retain a person’s dignity more effectively in any way?	Do you think you could retain the person’s dignity more effectively with help or support from outside?
		What sorts of treatments are available for frail older adults? Do you expect people to source these themselves or do they require prescription? Do you currently undertake any screening on older adults in standard care?	
Imagine you could assess [your own/a patient’s/the person you care for] frailty status via a set of questionnaires on a website. How would you feel about this? Would this be helpful?			
Imagine that you could train [your health/a patient’s health/the person you care for], in order to reverse frailty or to prevent it via a website. For example, by watching exercise videos on a website that show you how you can train your body to increase your strength. Would this be something you’d be interested in? Where would be the best place to offer these services (prompts: at home, at their local physical therapy centre or somewhere else)?			
What difficulties would you expect if treatments or interventions (such as health or exercise training) for frailty were to be introduced more widely? Do you think that is a good idea? What benefits would that have? What might be the problems with that (prompts: adherence, lack of trust, use of resources, worries about being labelled)?			
Is there anything else you would like to discuss?			

Discussions with older adults and caregivers were held in non-medical settings, so that participants could feel more at ease when describing their experiences, expressing their needs and complaints. Sessions were audio-recorded and conversations transcribed verbatim. Transcriptions and preliminary analyses were conducted in Polish.

### Recruitment strategy

Older adult participants and their caregivers were recruited purposively from general practice health clinics across the Lower Silesia District. They were invited to take part in the study by their family doctor at their planned practice visit. Older adults were required to be aged 65 years or over and fluent Polish speakers. Healthy older adults had no conditions of frailty or pre-frailty as assessed by a General Practitioner (GP) while frail older adults were assessed as having those conditions. Frailty assessments were undertaken by the general practitioner in the surgery. Frailty was defined as a clinical syndrome in which three or more of the following criteria were present: unintentional weight loss (10lbs in the past year), self-reported exhaustion, weakness (grip strength), slow walking speed, and low physical activity [8]. Caregivers were required to be taking care of a frail older adult on a regular basis, although they did not have to be residing with them. Health professionals were similarly recruited from the general practice health clinics of the Lower Silesia District. They were contacted through professional networks, in person, by telephone and via email. Social workers were all employees of the Regional Social Welfare Centre from Wroclaw. They were informed about the study by the Director of the Centre and were given time to take part if they wished.

Both health and social care workers were required to have at least two years’ experience in their respective fields. Attention was given to balance gender, age, role, and the type of professionals. In total 63 participants were invited and 44 accepted the invitation and took part in the study (response rate 70%). The main reason given for non-participation by all groups was a lack of time.

The final sample size was determined by the research design, a pragmatic outlook and availability of participants. The level of agreement and similarity within and between individual accounts, focus groups and stakeholder groups was very high. We explored individual as well as group perspectives, and the same comments and themes were raised throughout. Six researchers were involved in separately analysing and triangulating translated data. Thus while we cannot conclusively say that saturation was achieved, we are confident that the results are accurate and representative of the various stakeholder groups.

### Study duration and schedule

Recruitment started after Ethics approval and took two months. The focus groups were held between October 2015 and January 2016. Separate focus groups for all stakeholders were arranged. The focus groups took place in a non-clinical, seminar room at the University of Wroclaw, except for the meeting with social workers, which took place in a regional welfare centre. Transcriptions were made soon after the last focus group, and were followed by analyses.

### Ethical issues

The research was performed in accordance with the Declaration of Helsinki for Human Research of the World Medical Association and was approved by the Bioethics Commission of the Medical University in Wroclaw, Poland; Approval No. KB-502/2015. All participants had the opportunity to review information about the study and gave written informed consent. Information was written in a clear, standardised format. Participants were not reimbursed for their efforts. In order to maintain confidentiality, participants’ names and personal information were excluded from the transcripts and all quotations were anonymised.

### Data analysis

Interviews were transcribed in Polish by an IT specialist of Wroclaw Medical University, Poland and a GP (DK). Preliminary themes were noted which related to the accessibility of health and social care services. The data were synthesised using framework analysis [35]. This was performed by a psychologist with experience in both qualitative analysis and frailty (HG), a GP (DK) and a nurse (KS). Framework analysis is a five stage process which involves: familiarisation with the data; identifying a thematic framework; indexing responses; reviewing and revising the framework; and mapping and interpretation of themes. Following initial familiarisation with the data, Levesque, Harris, & Russell’s [32] theoretical framework was identified as most appropriate to make sense of the data. Data (including relevant participant quotations) were categorised and indexed into an Excel spreadsheet according to five dimensions of accessibility to care: approachability; acceptability; availability and accommodation; affordability; and appropriateness [32]. The framework was reviewed and revised, understanding of the quotations and translations was checked and the framework reordered as necessary. The authors then developed the explanatory account (narrative) for this paper from the revised framework. The Critical Appraisal Skills Programme (CASP: 2017) Qualitative Research Checklist was used to guide the conduct of the methods and to structure the presentation of findings.

## Results

The study involved 44 participants: frail patients (FP = 9), non-frail patients (NFP = 11), patients’ caregivers (PC = 6), health care professionals (HP = 9 including 6 general practitioners and 3 district nurses) and social care workers (SW = 9).

Five dimensions of accessibility to services are described: *approachability*, *acceptability*, *availability and accommodation*, *affordability*, and *appropriateness* [32]. Each theme is presented with example translated quotations. Quotations are attributed by participant group and participant number.

### Approachability

The dimension of approachability relates to the fact that people facing health needs can identify that some form of services exist, can be reached, and have an impact on their health. The corresponding ability required from the population is the ability to perceive that such a service exists.

Participants’ perceptions were of an opaque system that was complex and difficult to navigate. Both older adults and caregivers described the effort required to find their way around services and gain advice. They suggested that services should make themselves known and be more transparent, which would contribute to the service becoming more approachable. In particular, there was a strong need for the provision of more detailed information regarding available treatments and services, as well as psychological assistance, specifically for caregivers. During the discussion, one of the participants identified that she was not aware of a particular service, suggesting that this service was not visible, and therefore not approachable.

*“I, for example, had no idea that such person existed at all, as you said, a social worker […] Probably we don’t know that such a person exists and that person has no idea of our existence.” [NFP11]*

Although this person was a non-frail older adult, this lack of knowledge about services was concerning and might have prevented this individual from benefiting from this service in the future. Other caregivers also described the difficulties they faced in navigating the dual health and social care systems and in determining whether the service they required existed. In effect, they described a feeling of ‘not knowing what they didn’t know’, that is, they were unable to determine where the gaps were in their knowledge of the range of health and social care services provided and the type of support they could expect from professionals. This in turn made it difficult to determine how best to fulfill the care needs of older adults.

*“all these voluntary services and so on. But we have to know about it all.” [PC5]*

*“It shouldn’t be this way that I’m supposed to search, make phone calls and ask for training or something else, we should simply obtain this information.” [PC5]*

One of the proposed solutions to this lack of transparency was the adoption of a care coordinator as a new position in the health care system. People spoke of the need to create a “*liaison” [HP6]* between the health and social care systems and to be a conduit for information between services and patients.

*“this liaison, it should be someone who has knowledge about what kind of people she or he has in her or his area because these ladies from social care are ladies who work in this way that they come, do the shopping, if washing is needed the wash, they cook and they go. […] Whereas, they don’t do an interview, they make no reconnaissance in this area, whether something else is needed when it seems they should do that.” [HP1]*

For this individual, the coordinator should have a broader responsibility to the older adult than the social carers, they should be perceptive to new needs or requirements and act as a facilitator for, and advocate of care. Other participants also hoped that the coordinator would be able to guide them through the complexities of the legal system, as well as the medical system.

*“Yes, it should be exactly as you’re saying, a medical coordinator and a legal coordinator. It’s because there are some legal matters that need to be taken care of.” [PC6]*

In summary, this theme suggests that there are challenges associated with the approachability of services in Poland. Stakeholders described issues relating to knowledge of the existence of services and the difficulties they perceive in accessing those services.

### Acceptability

This dimension relates to the cultural and social factors affecting services. It examines whether people in a particular population (e.g. age, gender or social group) will accept the service and whether they judge it appropriate. The corresponding ability of the population to seek health or social care also relates to the concept of personal autonomy and the capacity to choose to seek care. Clearly in the case of frail patients, some will have the capacity to seek care while others may require an advocate, perhaps a family member, or a professional social care worker.

Stakeholders raised the idea of the need for psychological care, primarily for caregivers. Culturally, there appeared to be an acceptance of the need for psychological support, notably during caring episodes and also following bereavement. People spoke openly about their need for mental health support. Those who had experienced psychological services, described them as beneficial. One family caregiver described the inability to cope with her emotions, and the adjustment in her lifestyle after taking on the responsibility for caring for her mother:

*“Well, sometimes I made serious mistakes in the beginning, I couldn’t reconcile myself to it, I reacted, a bit, you know what I mean, emotionally, aggressively […] it is [caring for parent] a very big burden for me […] and I’m already looking for some help for myself…yesterday, I went to a psychologist to talk because I simply can’t cope with it.” [PC2]*

This extract describes the sense of anguish and anger the caregiver felt during the transition period from independent adult to caregiver, and their struggle to create a new sense of self, and to identity as a carer. However, it also demonstrates an adaptive coping strategy in that the participant identified their inability to cope and had the capacity to take positive steps to find help, and found that help acceptable.

Other caregivers also raised the idea of psychological support, specifically following bereavement. One carer, who had taken care of her mother for a long time spoke about her difficulties.

*“I want to say that later when you have nobody to care for, the first month you don’t even know you’re alive or not […] I am still not completely ok.” [PC5]*

Although the carer was grieving for her mother, there is an implication here that she is also grieving for her identity as a carer, in that she has lost her sense of purpose, or sense of self. Together, these two extracts address the need for psychological support, suggesting that stakeholders in older adult care and frailty find these types of services both acceptable and appropriate.

In terms of the acceptability of specific health care services, participants focused on their need for specialist health care, in particular, rehabilitation services. Rehabilitation services are a very important element of frailty syndrome prevention and therapy. The demand for such services is high and the participants were aware of the positive and real impact of these services on health, including improving physical fitness and independence. One caregiver who paid privately for a physiotherapist for her mother emphasised the effects of treatment:

*“This physiotherapy really came in handy, it was fantastic, it’s really hard to believe it. She [the physiotherapist] started to come twice a week, train, do a little massaging and mum is a lot fitter and she even started to exercise with me willingly because when I wanted to exercise with her she didn’t believe me and didn’t want to.” [PC2]*

As well as demonstrating a change in physical health, the above extract alludes to a change in beliefs about the acceptability of exercise as well as a change in self-efficacy through the willingness to take ownership of one’s health and to take part in additional physical activity.

Other social factors may also affect the capacity of people to choose to access services. Waiting times will be described in the next dimension of access to services - *availability and accommodation*, but long waiting times may place increased social burdens on patients and caregivers. The inconvenience and exasperation of waiting in line for a year for a scheduled doctor’s visit while struggling daily with the burdensome symptoms of coexisting chronic illnesses may contribute to loss of wellbeing and quality of life, as well as depleted mood and even the occurrence of depressive symptoms, in both frail older adults and caregivers. Further, the reduced economic status of many Polish older adults and their inability to allocate resources for private treatment may also increase symptoms of frustration and depression.

### Availability and accommodation

This dimension examines the ability to reach services, i.e., whether health services are available and can be reached physically and in a timely manner. Despite a common belief amongst participants in the power of physiotherapy and rehabilitation services, according to frail participants, the availability of those services, is difficult.

*“I think that rehabilitation would be a great help. Only that receiving rehabilitation is close to being a miracle.” [FP3]*

*“I would like to go to a [rehabilitation] meeting. I would go, because my legs hurt, and maybe I could lose weight, because that's also a problem.” [FP7]*

Similarly, other services which were perceived as valuable and necessary by stakeholders in terms of effects on their health were also difficult or impossible to access, specifically psychological support and periodic respite care. There was a strong belief that psychological support was ‘*very important’ [HP6]* for both older adults and caregivers but an understanding that these services were in very short supply, a view which was confirmed by a health care professional:

*“I won’t even mention psychological assistance, which, of course, is very important, but in Poland it is practically non-existent.” [HP6]*

The difficulty of availability here is that there is a limited provision of this specific service - psychological support - which outstrips supply.

Caregivers also indicated a desire for free periodic respite care, specifically to have time for themselves, either to have a chance to relax or devote it to solving other important matters, for instance, their self-care and personal health issues.

*“Would it be possible to implement a programme so that you could leave such a person but I think you need to pay for that, I don’t know if for a week ... So, you could leave that person for weekly rehabilitation and so that the person who cares for her or him could simply have a rest?” [PC6]*

However, one of the greatest difficulties expressed by participants relating to availability of health care services was the waiting time for a doctor’s appointment or scheduled surgery. Participants described how the normal waiting time for an appointment with a specialist is a few months, while it may take several years for scheduled surgery.

*“The Doctor refers me to a specialist. And the man tells me, someone tells me – please come back in 8 months.” [NFP 2]*

*“Today, I talked to my friend, younger than me, I was giving her my best wishes. I asked her how she was feeling because I knew she had had a problem with her hip for a long time. She tells me, I’m OK, you know. I told her I thought she had already been operated on. No, her appointment is in 2020.”* *[NFP7]*

Although participants were pragmatic when describing these long waiting times, there was also an element of frustration associated with the accessibility of specialist medical care, and a view that long waiting times were themselves a contributory factor in ill-health, a view which was described by a participant waiting to see a cardiologist.

*“I think that […] one of the principal causes of this frailty of ours is the present day doctor’s, medical care. I mean […] for example registering the patients for the next year.” [FP8]*

Certainly, the length of waiting times had an effect on quality of life and independence and was problematic in a number of cases.

*“I had been making efforts to arrange rehabilitation [for my wife] and when after waiting for a long time the rehabilitation took place my wife was no longer independent.” [FP4]*

In this extract, the participant describes an inexorable shift in health and dependency before the rehabilitation treatment could take place. The time lag between the intervention being suggested and delivered meant that the older adult had transitioned outside the boundaries of that specific intervention treatment window. Preventative medicine in older adults encompasses a range of interventions and some may indeed prevent an individual from tipping over into a more intensive (and consequently expensive) level of treatment or care plan. Unfortunately, the difficulties associated with waiting times were not just limited to health care services. Legal issues were also affected as described by this participant.

*“The doctor who was supposed to care for my mum said I should arrange my mum’s incapacitation.[legal arrangement to appoint a guardian to make decisions on your behalf,] So, we will keep her here for 3 days and you arrange that. It finally took half a year to arrange and then my mum passed away.” [PC5]*

In this instance, the participant described how suitable legal provision could not be made for her mother before her death. This participant raised concerns about obtaining the appropriate legal services and the cost implications of those services. They described how difficulties in communication and managing legal matters delayed care. There were other examples of communication issues between departments delaying care. One social worker described how the dissolution of the health and social care system into two separate ministries and the restructuring of care, had resulted in additional bureaucracy and a lack of communication which delayed the onset of social care.

*“In serious situations when there was a caregiving services division some years ago before it was liquidated, […] you would find yourself in a situation that for example there was a lady discharged from the hospital, cancer, […] and I came for the interview and I called them, send me a caregiver because there is this case and she is needed immediately. And they would send her. The decision has not been made yet.” [SW1]*

Sadly, there are human and moral costs here, in terms of the quality of life for older adults, as well as issues of availability.

Another issue related to availability and accommodation is the physical availability of clinics and staff. Given staff shortages, health and social care professionals are often located a distance away from their patients.

*“Whereas these ladies […] who are supposed to coordinate, they generally sit in the office and it is a great problem to get them to come to a community interview because they don’t have money for the lump sum, for gas. And the area is vast, the municipality is very big, so you need drive a lot.” [HP8]*

In this instance, we note that dimensions of accessibility to health care are not completely independent constructs. Here, the geographical range of the clinic, shortage of qualified staff and the costs involved for staff to travel long distances, affects the availability of the service.

The theme of availability and accommodation also relates to the characteristics of providers, for example in terms of their qualifications. Some participants spoke about their lack of satisfaction with the availability of doctors, and difficulties in maintaining continuity of care.

*“Here the problem is the turnover of the doctors. Since one doctor starts the treatment, grasps everything that is needed, then a year later she/he is gone.” [NFP2]*

Conversely, when continuity of care was present, it was recognised and greatly appreciated by the patients and caregivers.

The availability and qualifications of social care workers were of particular interest to the stakeholders. The social care system is struggling with the volume of older adults needing assistance and few people are interested in working in this sector. Consequently, care workers are scarce.

*“In [the Old Town] we currently have 4 girls who deal with care services and they have 70 persons each. This is a huge number. There are too few caregivers. In [name of city] when I was preparing this map of resources and needs, the thing is that in 2017 in [the Old Town] 25% of the society will be in post-production age.*^1^” *[SW8]*

Critically, the social care professional describes the scale of the problem in a sector of one city and the lack of availability of staff, as well as highlighting an ongoing and escalating crisis in the future. Concerns were also raised about the way in which care workers were recruited to the profession and trained. A professional caregiver should be properly trained and prepared for work with older adults and they should receive appropriate remuneration for it. Currently, this is not the case; participants reported that caregivers are selected and recruited almost randomly and have minimal training.

*“They are often unemployed people who we being the social workers send to the Social Welfare Centre: please try to get hired there. They can be completely unprepared for this.”* *[SW8]*

*“They [the social care workers] are hired through a ‘roundup’ because there are no caregivers, the caregivers don’t want to work.”* *[SW1]*

These extracts describe a social care system which has serious issues related to training and competence for care workers and safeguarding for older adults. Participants had a number of sensible suggestions to improve conditions for carers and their patients, including proper training and adopting other good practices.

*“I think we should start by educating the workers who are to deal with these problems and care of the elderly and communities.” [HP1]*

*“The UK system, […] their social care is very much specialised. Here we don’t have that at all […]. There you have social workers for mentally ill patients, there are social workers for patients with cancer. There, if you have some kind of problem with a patient, you call specialist services.” [HP8]*

Participants described how applying financial incentives, ensuring an employment contract (as compared to the current fee-for-task agreement) and appropriate training (and where appropriate specialist training) of the social care professional, could improve the situation for future cohorts of older adults.

### Affordability

This theme relates to the economic capacity of people to spend their time and resources to use health and social care services. Older adults, both frail and robust, relayed their frustration with the inequalities inherent in the current system of health care and their awareness of financial barriers to health, in the first instance relating to access to specialist examinations and doctor’s appointments.

*“When I am depressed or something else is wrong, I need a referral to a specialist and what? If you don’t have a hundred [100 zlotys] you sit [and wait] half a year [for an appointment with a specialist].” [NFP2]*

*“If it hadn’t taken paying 500 zlotys, we would have waited for a year [for an MRI scan after a stroke]. And in this way it is possible in a week’s time. Then this is where the problem is.” [NFP2]*

As illustrated by these quotations, timely access to specialists is perceived as only being available for people with sufficient financial resources. In the latter quotation, the participant described the alternatives of having to wait a significant length of time for a necessary scan after a serious, life threatening medical condition or paying privately to have the scan done immediately. So, it would seem there is capacity within the system to provide resources, they are available, but they are just not accessible to all. This suggests a system of health inequality. We know that ill health is related to poverty and this is particularly significant here. Given that many frail older adults are in receipt of fixed incomes through pensions and other benefits, these resources may not be sufficient to fund all their health or social care, which suggests that needs or conditions may not be treated at the optimal time, or worse, may be going untreated or unmanaged. Certainly, this was a viewpoint suggested by one of the frail participants in the study.

*“if it’s a private visit then you have to pay and not all of us can afford that, for example me, I can’t afford it.” [FP8]*

And social workers echoed this viewpoint and confirmed the inequalities endemic in the system:

*“There are no [free] health care centres in [town name]. We have the [name of centre] who charge 100 zlotys a day and another at [name of street] who charge 4,500 zlotys a month. And we have people who get 600 zlotys of their monthly pension. Where should they get the money from? I call the doctor, the head [of one health care institution] and he doesn’t ask me about the patient’s state, but how much money she/he has. There is no cooperation whatsoever! Because it is all about the money.” [SW1]*

In this extract, the social worker described a disconnectedness between the priorities of the stakeholders involved in the care of the older adult. On one hand, the social worker is attempting to obtain the most suitable care for their patient, while on the other, the professional in charge of the clinic, ironically a doctor, is ‘blocking’ treatment and prioritising resources and finances over the individual’s health. The extract demonstrates the challenges faced by both professionals in their daily work but also describes an arrangement whereby the social worker is acting as an advocate on behalf of the financially disempowered and potentially vulnerable older adult. A further example of a disconnect between doctor and patient and the social carer as advocate is also evident in the way that this social care worker describes the way in which doctors prescribe medications.

*“The doctors are very often unaware of the way in which these patients live. What is their financial situation? There are some medications that are exorbitantly expensive. It is us who tell these patients: please ask the pharmacist, maybe there is a cheaper alternative. These people don’t use the medications because they just can’t afford them. They come to us and ask for financial assistance. Therefore, cooperation with the physicians, when it comes to the elderly, is for me the most important thing.” [SW8]*

This participant details a lack of understanding and awareness on behalf of the doctor of the patients’ individual circumstances and the affordability of prescribed medications. Critically, they describe how financial barriers result in cost-related non-adherence to the treatment plan which has the potential to affect important health outcomes. Here, this social worker is also advocating a closer and more integrated working relationship between the health and social care professionals such that the whole person context is taken into account when developing a treatment regime.

The social carers also drew attention to difficulties and economic barriers in relation to accessing long-term inpatient care.

*“There are vacant commercial places, but they cost 4 thousand [zlotys]. Whereas, you need to wait half a year for a subsidy from the National Health Fund.” [SW6]*

*“Half a year up to one year.” [SW1]*

*“Half a year? A year or one and a half years.” [SW3]*

The key issue here is a shortage of suitable accommodation and a lack of institutions that can provide a ‘round-the-clock’ service for older adults. With limited supply and increasing demand, inevitably the costs associated with private nursing homes are very high and this is exacerbated by long waiting times for subsidies from the National Health Fund.

One of the key issues within this theme of affordability was that families of frail older adults were willing and prepared to support their loved ones. However, they are not themselves supported to do this through financial means such as a carer’s allowance.

*“For centuries we have been brought up in a traditional, multi-generation family that took care of the elderly. […] let’s pay, we have these benefits for the caregivers, let’s pay for it in some way. Maybe I will be able to choose whether it pays for me to continue working or care for my parents. I will choose one or the other.” [SW3]*

This participant describes how providing benefits and assuring financial support for family members with caring responsibilities, would provide them with a range of options concerning the most appropriate care for their loved ones; with such support, it would be viable to stay at home and care for their loved ones, rather than relying on social care.

### Appropriateness

This dimension relates to the fit between services and the clients’ needs. One concern of participants was that the current health care system is ‘firefighting’, i.e., focusing only on the provision of emergency treatments in urgent cases.

*“We only focus on solving emergency problems […] those patients who require some afterthought and a more integrated care simply slip by. Because there is no chance, there is even no time. Physically there is no time to devote at least some of your attention to them during work. And I also think it is because of the scarcity of funds.” [HP8]*

Here the health care provider explains how a lack of resourcing means that people with complex health care needs may be ignored in order to concentrate efforts on the most acute cases. This focus on emergency or ad hoc health care was echoed by other participants.

Conversely, participants described how the social care system did not function well in a crisis. Stakeholders described how many older adults are simply allocated a caregiver by default, whoever is available at the time, and described how these personnel are often poorly trained and low paid. Social workers also mentioned that they have no direct influence on matching older adults with their caregiver. Similarly, social workers are unable to change an older adult’s caregiver if a relationship breaks down, and are unable to call them off where needs change.

*“When we, as social workers, go to implement these services then we no longer have any influence on the choice of the caregivers […] It is because the entity which realises the services is already another institution.” [SW4]*

This social worker describes how the allocation of resources from various different bodies and institutions adds layers of unnecessary bureaucracy to the social care system and means that social workers are unable to communicate effectively with caregivers to ensure that the needs of the older adult are properly met. Critically, the social worker participants hoped for a streamlined social care system with a reduced level of bureaucracy. They pointed out that their willingness to assist people is often hampered by complicated and time-consuming bureaucratic procedures.

*“Going to see the client and carrying out the community interview is only one tenth of the work. The interview is 16 pages long.” [SW5]*

*“The impression was that someone wanted voluntary services, someone wanted them fast. Two years. I did that in [city district] literally, me and a friend had been thinking for one and a half years how to do that. How to go through personal data protection, the paperwork and all […] we want to answer people, citizens’ needs promptly. It isn’t easy, we say that straight away.” [SW5]*

In these extracts, participants describe how bureaucratic and administrative processes affect the quality and timeliness of care for older adults. However, there was a willingness among participants to improve services and to assist people, and they described working within the regime to try to speed things up. Some participants expressed a desire to return to the caregiving services division, which had existed previously and which participants felt worked well in an emergency situation.

Participants described the need to introduce legislation to enable social workers and caregivers to care for older adults ‘around-the-clock’ (24 h a day, 7 days a week).

*“There is 10% of such people who would require care in the night hours […] The Act provides for a maximum of 8 hours […] This is ad hoc care and not 24/7 care.” [SW1]*

*“In fact they [social care workers] work from 8 a.m. to 4 p.m.” [SW4]*

This statement suggests that current legislation protects employees and workers over vulnerable adults and implies that some vulnerable, dependent older adults are unable to access care for around 16 h per day when residing in their own home. This is a situation which has serious ethical implications and may mean that older adults are placed in nursing homes, which may not be the most appropriate residence for them, and which may be more costly.

The essence of the current functioning of care for frail older adults in Poland could be summarised by the statement of one of the participants.

*“There is a seed of a system, there are some nurses, there are some caregivers, but this [system] is still not working, or is unable to work.” [NFP5]*

In light of the shortcomings of both health and social care systems and the difficulties older adult patients face in satisfying their health and social needs, participants suggested a need for a new type of system, an integrated care system with comprehensive staff training and a multidisciplinary team.

*“I think we should start by educating the workers who are dealing with these problems and care of the elderly and communities. And then we could try to create, for example, multidisciplinary groups, medical and social, and I would ask here for a person such as maybe a psychological aid. It would be a cool team, a social and therapeutic team and maybe then we would be able to do something.” [HP1]*

Participants also expressed an expectation that major legal and organisational changes would be required from the national institutions to manage a welfare state in the face of an ageing population. They recommended the restructuring of the currently disparate health and social care systems, as well as a move towards more comprehensive preventative medicine including more frequent home visits and preventative screening by health practitioners including family doctors and nurses.

Participants asked questions about public finance and resource management, suggesting that these should be reviewed to increase the effectiveness and efficiency of the public sector in order to guarantee appropriate medical and social care to older adults.

*“First of all, on our part, withdrawal from the so-called ‘radar medicine’, which means focusing on ad hoc problem solving in favour of comprehensive care of the patient [..] Second, total reformulation of the social care system, a really deep reformulation.” [HP8]*

In terms of both health and social care, participants felt that the new system must ensure continuity of care, by more ‘permanent’ physicians and nurses, as well as caregivers. The aim of this was to ensure that older adults felt secure with their health care provider or caregiver and were able to build a truly cooperative relationship. Within this idea of positive, trusting relationships, people spoke about the importance of assistance from neighbours and the need to formalise some of these necessary but informal caring relationships.

*“Within the caregiving services someone from close-by, a neighbour exercised care over a given person and she or he would be paid for that. And it would be great.” [SW3]*

*“That is because that person was close by [SW4]. You didn’t even have to look for her or him, she or he was there and could even come along at night.” [SW1]*

Social workers in these extracts described their suggestions of how neighbourhood carers might be paid for their assistance to older adults, and how well the relationships might work. They noted that there is an element of trust, as the person is local and sometimes known to the older adult, and also how that given that they are located nearby, they could help when required throughout the day, rather than just at set times.

## Discussion

Due to their complex and continuously changing health and social care needs, frail older adults require access to a wide range of services over a long period of time [36]. However, in this research participants clearly described the difficulties they had encountered when accessing health and social care. These difficulties included a lack of knowledge regarding the existence of some services, under-supply of services, long waiting times for specialist care and rehabilitation services, geographically remote clinics, staff shortages, a lack of trained and competent social care professionals, high staff turnover, shortages of long-term inpatient care, economic barriers to care and inequity in care standards. In terms of the appropriateness of the current system, participants described issues related to a high functioning emergency health care system which was unsuitable for patients with complex needs and a low functioning emergency social care system, where the bureaucratisation of systems caused serious delays in providing services to the vulnerable. Fundamentally, there were also legislative issues which meant that the most vulnerable older adults were unable to be cared for around-the-clock. In essence, stakeholders conveyed that the Polish system is designed for healthy adults with acute illnesses, not for an ageing population with complex health and social care needs. Certainly, this can be confirmed to some extent by statistical data. In Poland there is a shortage of geriatric medical care (encompassing medical professionals as well as specialist geriatric wards and clinics) for older adults [6]. Moreover the ratio of dependent inpatient care services per 1000 head of capita is one of the lowest in the European Union (25.0 workers in Poland vs. 46.7 in the UE-27 states in 2014) [21].

There were a number of issues relating to availability and accommodation of access to health and social care. Participants in this research placed particular emphasis on staff shortages in both the health care and social care sectors and the scarcity of financial means allocated for care. The high workload of physicians means that resources are directed in an ad hoc manner at urgent and emergency cases. Further, there is a high turnover of family medicine specialists which means that continuity of care is disrupted. In turn, social care workers are under increasing pressure with rising numbers of patients, payment on a fee-for-task agreement (i.e. output work) and low remuneration. All of which contributes to a shortfall in the number of candidates wishing to take on this type of work. Some suggested solutions to these difficulties include introducing appropriate financial incentives for family doctors or ‘pay-for-performance’ schemes linked to the achievement of specific clinical and organisational targets, similar to the UK model. While these systems may induce this group of professionals to take over the role of the “gatekeeper” and as a consequence prevent offloading the costs to higher levels of care [37], they have also been linked to an increased administrative burden and bureaucracy [38] which the stakeholders in this study were keen to reduce and avoid. We suggest that promotion of social care as a positive career choice with appropriate remuneration, alongside employment contracts and appropriate training would go a long way to solve many of the problems but the mindset of both policy makers and private care providers, as well as potential career carers, would need to change. This would ensure that the quality, competence and professionalism of social care staff would improve, the potential pool of social care candidates would increase, and continuity of care would benefit.

Another critical access issue was the availability of long-term care (LTC) and challenges associated with accessing it. From a public health perspective, a solution to LTC requirements is critical. As yet, Poland has not established comprehensive national LTC programmes, relying on informal caregivers combined with a fragmented mix of formal services that vary in quality and by location [39]. Responsibility for LTC in Poland is divided among the central government, governmental health agency, governmental labour and social agency, and territorial self-government [40]. The decentralisation of the government and public administration has led to a lack of ownership in the development of a strategic LTC plan. Currently, responsibility for organising LTC resides with local governments, while the material responsibility for the form and contents of care and its financing belong to the health care sector, with the consequence of difficulties in action coordination [21]. In addition, the integration of LTC services also face problems from the integration of institutions that operate on the margins of the health care system with institutions that operate within the social assistance scheme, and in the integration of residential care and home care [40]. We suggest that the integration of the health and social care systems could result in better cooperation between the professionals of both sectors. The first stage of integration might consist of multidisciplinary team meetings to share information about the patients’ health and social situation and to develop suitable care plans. The introduction of integrated IT systems would also assist in ensuring that all members of the therapeutic team have insight into the treatment history and care of the patient. These are not inconceivable goals, other countries, for example the United Kingdom have implemented similar systems as components of care in an attempt to improve patient outcomes and reduce delays.

In terms of an appropriate system, the need for a coordinated and continued medical and social care system was paramount, and was tangibly expressed by all participants in this study. Some of the proposed solutions to ensuring satisfactory medical and social care for all older adults included the ‘new management’ of public funds, ensuring around-the-clock care for older adults from a community nurse, reducing the level of bureaucracy in the social care system, formalising assistance from neighbours, ensuring financial assistance to family caregivers and greater cooperation between health and social care staff.

Financial support to informal caregivers was a particular concern to participants. In light of an increasing number of older adults in society, a greater number of caregivers will be required, and therefore it makes sense to develop appropriate ‘future-proof’ legal and financial mechanisms that will enable caregivers to reconcile professional roles with caring responsibilities. At present, in order to receive a caring allowance, a caregiver must not be in employment [22] but the Polish pension system is such that caring responsibilities are not recognised as reckonable service and thus, informal caregivers are effectively penalised for giving up work to care for loved ones. Further, there is no assistance in helping people re-enter the labour market when their caring duties are over.

Another critical issue for caregivers was the physical and mental toll caring takes on them. In the present study, informal caregivers expressed a need for much greater psychological and informational support as much as periodic relief from performing difficult caring responsibilities. Here there was a suggestion that services were difficult to approach, participants either did not know that they existed, or did not know how to easily navigate them. However, in terms of acceptability, there was an acceptance of the value of such services from participants. The value of psychological services is also borne out in the literature, both for caregivers and older adults. A systematic review [41] described the three main types of support needed by caregivers: respite, psychosocial support and information, and communication technology support. The authors concluded that an integrated support package tailored to the individual caregivers’ physical, psychological, and social needs should be preferred when supporting informal caregivers of frail older adults. Further, evidence from eleven randomised controlled trials suggests that supportive interventions may help reduce caregivers’ psychological distress [42]. These authors suggested that practitioners should enquire about the concerns of caregivers and should consider that they may benefit from additional support. In another study [43], caregivers’ depression, stress, or burnout increased the risk of institutionalisation for the older adult. While cost-effective caregiver support policies can reduce the demand for expensive institutional care [44]. Thus, we suggest that any health and social care reforms should ensure that caregivers are given appropriate support, both for their own health but also as a potential method of cost saving. Certainly, the cost effectiveness of caregiver support versus ongoing institutionalisation would be worth exploring in future research.

One role which might assist with the approachability and the appropriateness of services was that of the care coordinator. Although the role of a care coordinator was largely dismissed by European health care policymakers as unnecessary [45], the members of all the stakeholder groups in this study indicated the need for a care coordinator as a new, and desirable institution in the Polish care system. The coordinator would be a trusted mediator between the physician and patient and exercise the role of a “liaison” between the health care and social care systems. The envisaged role of the care coordinator would be to assess gaps in health care and to develop a personalised care plan to address the care needs of the participant. Care coordinators might also confer and collaborate with medical providers, review the use and appropriateness of, and adherence with, prescribed medications, accompany individuals to their medical appointments if needed, provide patient and family education, and assist with referral to community resources as appropriate [46]. Certainly, studies have shown that case management is beneficial. For example, in the United States, authors [46] demonstrated that case coordination and subsequent discharge planning reduced hospital admissions in high risk older adults on Medicare (over 70% of whom were aged 75 years and over) and $7.7 million in cost savings, as well as improving the uptake of laboratory tests and surgery visits. Conversely, other authors [47], suggested that the Walcheren Integrated Care Model, which involves care coordination, was not cost-effective, and moreover, the costs per quality-adjusted life year (QALY) were high (an average of 412,450 euros per additional QALY)**.**

Similarly, in another study [48], a 1-year intervention (*n* = 150 frail patients) was carried out by nurses and physiotherapists working as case managers, who undertook home visits at least once a month. Authors showed that there were no significant differences between the intervention group and control group for total cost or two measures of quality-adjusted life years. The results can be explained by the fact that the intervention group had significantly lower levels of informal care and help with instrumental activities of daily living (IADL) both as costs (Euro 3927 vs. Euro 6550, *p* = 0.037) and provided hours (200 vs. 333 h per year, p = 0.037). However, other studies have demonstrated that care planning and coordination by a case manager resulted in improvements in older persons’ subjective well-being. [49] Further, that older people receiving a comprehensive continuum of care intervention, including a care coordinator, perceived statistically significantly higher quality of care on items about care planning compared with those receiving the usual care and had increased their knowledge of whom to contact about care/services, after three and 12 months [50]. Certainly, given the strong desire of the stakeholders to adopt this model of assistance with health and social care, it may be worthwhile exploring in other Polish populations in future research.

Lastly, the affordability of services was a significant issue for participants. Although there is the premise in Poland that health care is free to the most vulnerable in society, in practice, many services are oversubscribed and have long waiting times and so to resolve issues more swiftly, private health care is common. However, in effect this is creating a two-tier system in that health care is only available to those who can afford it. Such a system has human, moral and ethical implications. In order to improve this system, there should be a renewed focus on the integration of health and social care services, investment in preventative measures in primary care, and a change of focus from outmoded bureaucracy to person-centred care which is responsive to the changing needs of the Polish community.

### Strengths/limitations

The heterogeneous sample with five groups of stakeholders enabled a comprehensive, multidisciplinary assessment of the accessibility of the health and social care systems in Poland and a broader look at the changes required by stakeholders in the future. Stakeholders demonstrated a significant level of agreement about the key access issues for frail older adults including timely, accessible and affordable care for all; continuity of care; and significant improvements in social care staff recruitment and training. Although this article only concerns access to health and social care in Poland, the results may also be useful in countries with similarly functioning systems, for example decentralised health and social care systems with low health care capacity.

## Conclusion

There can be little doubt that a rapidly ageing population generates complex access requirements for health and social care at both the individual and community level. Such changes in age distribution accompany a significant increase in the prevalence of chronic diseases, frailty, and disability, involving greater expenditure of resources and higher utilisation of community services. This results in an urgent need to build effective care systems for older adults as well as support networks for their families. Based on our findings, we make recommendations (see Table 2) to act as pragmatic guidance for the reader interested in improving their health and social care system. To summarise, for frailty care to be properly accessible to older adults in Poland, health and social care services need to be integrated in order to reduce bureaucracy and increase the timeliness of treatment and care. Further, the recruitment strategy and training of social care professionals should be reviewed to build capacity and competence within the profession.

**Table 2:** Recommendations to Improve Access to Health and Social Care Systems in Poland^a^

Access Issue	Recommendations
Approachability	Services should be transparent about what they offer, when and to whom. Where services are not transparent or easily navigated, a care coordinator (liaison or advocate) should be available to help caregivers and older adults to access health and social care services.
Acceptability	Psychological support should be available for caregivers, both during the caregiving activities and after bereavement. Rehabilitation services are in short supply but may result in changed attitudes towards exercise and ownership of individual’s health where provided.
Availability and accommodation	Timeliness is critical when developing interventions for older adults. There needs to be a reduction in waiting times for specific treatments for older or frail adults, in order to prevent the need for potentially more intensive (and consequently expensive) treatments or care plans. Bureaucracy should be reduced and administrative systems streamlined to prevent communication failures and significant delays in medical treatment or provision of social care for frail older adults The availability of low or no cost respite care should be investigated. There should be appropriate training and remuneration for professional caregivers.
Affordability	Doctors should recognise the reduced financial situation of some older adults and consider how that situation might affect adherence to (paid for) medical treatment. There is a willingness among some participants to care for older relatives at home with limited financial support. The cost effectiveness of financially supported home care versus existing alternatives should be explored.
Appropriateness	The current health care system provides high functioning emergency provision, which is unsuitable for patients with complex needs. A new model of care should be evaluated which moves away from acute crisis management in older adults and considers a more integrated and holistic health pathway. The current social care system is low functioning in an emergency situation and bureaucratisation causes serious delays in providing services to the vulnerable. There should be a change in legislation in order to provide effective social care for older adults outside normal office hours.

^a^Based on Levesque, Harris, & Russell’s [32] theoretical framework of access to health care

## Abbreviations

CASP: Critical Appraisal Skills Programme
EU: European Union
FP: Frail patients
GP: General Practitioner
HP: Health care professionals
IADL: Instrumental activities of daily living
LTC: Long term care
NFP: Non-frail patients
PC: Patients’ caregivers
QALYS: Quality-adjusted life years
SW: Social care workers

## Footnotes

1 Post-production age in Poland is over 65 years for men and over 60 years for women.
